# A fatal intractable ventricular fibrillation triggered by improper use of Xiaozhong Jiuwei Powder: integrated LC-MS chemical profiling unveils the cardiotoxic culprits

**DOI:** 10.3389/fmed.2025.1682854

**Published:** 2025-10-27

**Authors:** Halidan Abudu, Zhaofei Yang, Lu Zhang, Dilidaer Sidike, Liqiong Guo, Haojun Fan, Ayixiamuguli Wubuli

**Affiliations:** ^1^School of Disaster and Emergency Medicine, Tianjin University, Tianjin, China; ^2^The Key Laboratory of Xinjiang Endemic and Ethnic Diseases and Department of Biochemistry, Shihezi University School of Medicine, Shihezi, China; ^3^Department of Emergency, People's Hospital of Changji Hui Autonomous Prefecture, Changji, China; ^4^Department of Oncology, The First Affiliated Hospital of Xinjiang Medical University, Ürümqi, China

**Keywords:** Xiaozhong Jiuwei Powder, cardiac arrest, UHPLC-Q-Exactive-Orbitrap MS, Aconitum alkaloids, toxicity

## Abstract

**Introduction:**

Acute poisoning from toxic substances significantly endangers human health, with poisoning-induced cardiac arrest representing a critical public health concern. Xiaozhong Jiuwei Powder (XZJWP), a traditional Chinese medicine (TCM) formulation used in Mongolian Medicine, is known for its antipyretic, analgesic, and anti-inflammatory properties. However, improper use of XZJWP can lead to severe adverse effects.

**Methods:**

In this study, we report a fatal case of refractory ventricular fibrillation in a patient admitted to our hospital in autumn 2023, following the improper use of XZJWP. Despite intensive treatment, the patient succumbed to the condition 1 week later. To identify the toxic chemicals responsible for this outcome, a comprehensive chemical profiling of XZJWP was conducted using UPLC-Q-Orbitrap-HRMS.

**Results:**

Our analysis identified 292 compounds, categorized into 24 major groups, with several toxic alkaloids detected. Notably, diester-diterpenoid alkaloids (DDAs), including aconitine, mesaconitine, and hypaconitine, were identified as key toxic compounds known to cause neurotoxicity and cardiotoxicity.

**Discussion:**

These findings warrant caution in using XZJWP and other aconite-containing TCMs, emphasizing the need for public education on safe use and improved clinical management of related poisonings. The study provides crucial guidance for healthcare professionals in diagnosing and treating such toxicities.

## 1 Introduction

Cardiac arrest induced by poisoning is a significant public health concern, with epidemiological characteristics that have garnered global attention. Recent studies indicate that poisoning is a notable cause of out-of-hospital cardiac arrest (OHCA), with distinct incidence and prognosis compared to other etiologies (1–3). For instance, a 15-year national study in Sweden (2007–2021) revealed that poisoning accounted for 5.2% of non-cardiogenic OHCA cases (4). These patients were typically younger, predominantly male, and had lower rates of witnessed events and shockable rhythms. Similarly, a population-based study in South Korea highlighted that poisoning-related OHCA constituted a considerable proportion of non-cardiogenic cases, with improved survival rates observed in drug overdose-related OHCA (5). These findings underscore the diverse epidemiological patterns of poisoning-induced cardiac arrest across regions and populations, emphasizing its public health impact. Poisoning events, including drug overdoses, pesticide exposure, and ingestion of toxic substances, can lead to cardiac arrest, necessitating a comprehensive understanding of its mechanisms and the development of effective prevention and treatment strategies.

Xiaozhong Jiuwei Powder (XZJWP), a traditional Mongolian medicine, is widely used for its antipyretic, analgesic, and anti-inflammatory properties (6) and is a topical preparation. Clinically, XZJWP can be used to treat acute mumps (7, 8), lymphadenitis, subcutaneous and deep abscesses (9), dengue fever, redness, swelling, heat, and pain, rheumatic (10), and cold paralysis, and joint pain (6, 11). Composed of nine herbal ingredients—*Euphorbia pekinensis* Radix, *Rheum palmatum* L., *Potentilla discolor* Bunge, *Polygonatum odoratum* (Mill.) Druce, *Curcuma longa* L., *Acorus calamus* L., *Aconitum kusnezoffii* Rchb., *Asparagus cochinchinensis* (Lour.) Merr., and *Rheum pumilum* Maxim. XZJWP has demonstrated efficacy in managing gouty arthritis, preventing PICC-induced pain or phlebitis, and treating mastitis and arthritis. Modern pharmacological studies have further validated its therapeutic potential, particularly its analgesic, anti-inflammatory, and gouty arthritis-treating effects (9, 10, 12–15), aligning with its traditional applications. However, despite its widespread use, the therapeutic material basis of XZJWP remains poorly understood, with limited research on its chemical composition (16). This gap highlights the need for systematic investigations to identify its bioactive components and potential toxicities.

Our study was prompted by a clinical case involving a patient who developed intractable ventricular fibrillation following the improper use of XZJWP. Admitted to our hospital in autumn 2023, the patient succumbed to the condition 1 week later despite intensive treatment. This tragic outcome underscored the urgent need to investigate the chemical composition and toxic components of XZJWP. The primary objective of this study was to comprehensively analyze the chemical profile of XZJWP using ultra-high-performance liquid chromatography coupled with Q-Orbitrap high-resolution mass spectrometry (UHPLC-Q-Orbitrap-HRMS). This approach aimed to identify the toxic chemical components responsible for inducing severe cardiac arrhythmias, such as ventricular fibrillation. By identifying these toxic compounds, our study seeks to provide a scientific basis for understanding and treating toxicity-related arrhythmias caused by XZJWP and other similar traditional medicinal preparations. This research not only contributes to the safe use of traditional medicines but also offers valuable insights for the clinical management of poisoning cases associated with their misuse.

## 2 Case report

A 25-year-old male patient was admitted to the hospital at 16:30 on October 2023 for vomiting and numbness of the lower limbs for half an hour after accidentally taking 30 g of the topical drug XZJWP. Half an hour before admission, the patient took 30 g of XZJWP, a topical decongestant, and experienced nausea and vomiting, with gastric contents, no coffee-coloured vomit (the amount of vomit was unknown), and a feeling of panic, general weakness, numbness of the lower limbs, and no other concomitant symptoms. He was sent to the emergency department of our hospital by a colleague. Patient accompanied by the staff on behalf of the complaint: half an hour ago due to double eye swelling and pain discomfort, self-service XZJWP powder 30 g (for external application of drugs) swelling pain symptomatic, then nausea and vomiting, vomiting, vomit once, vomit for the stomach contents (vomiting amount of specific is not known), feeling panicky, general fatigue, denial of headache and dizziness, denial of chest pain and chest tightness, denial of respiratory distress, denial of abdominal pain and diarrhoea, self-consciousness of numbness of lower limbs and weakness, colleagues found that immediately after the Emergency Department of the hospital for medical attention! He was admitted to the emergency department of our hospital with the diagnosis of drug intoxication. The patient had a history of syphilis infection, No history of extramarital sexual contact, no history of trauma or surgery, no history of smoking or alcohol consumption, no drug allergy, no history of drug abuse, no history of cardiovascular disease and no history of other chronic diseases.

**Figure 1:**
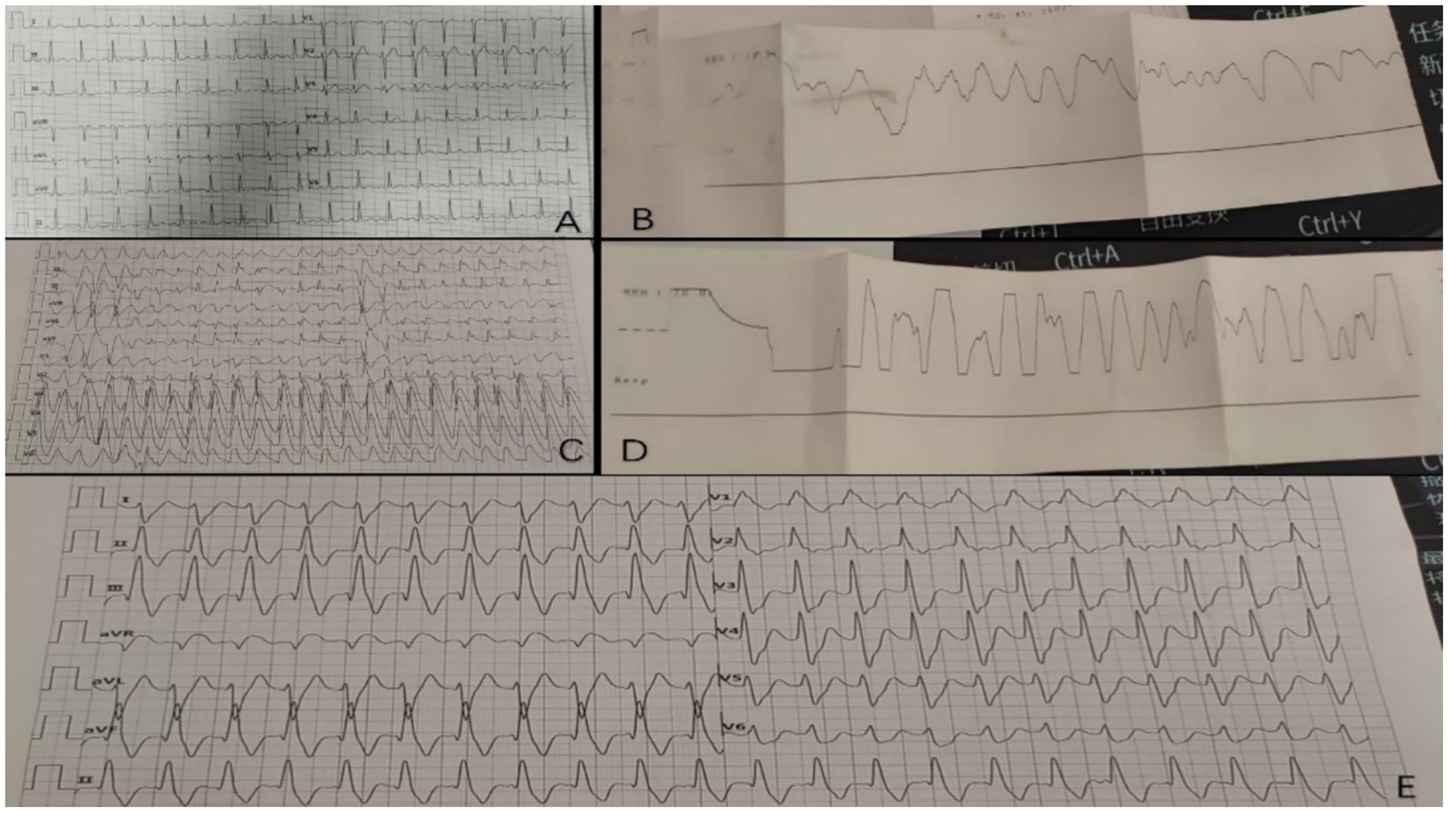
Electrocardiographic changes in patients before and after treatment. **(A)** Sinus tachycardia on EKG at admission; **(B)** Ventricular fibrillation on EKG at 17:23; **(C)** Ventricular tachycardia on EKG at 46 min; **(D)** Ventricular fibrillation on EKG at 63 min; **(E)** ventricular tachycardia on EKG at 21:54 when transferred to a higher hospital.

Physical examination on admission revealed: Vital signs: Body temperature 37 °C, respiration rate 21 breaths/min, pulse 107 beats/min, blood pressure 95/58 mmHg. General condition: Conscious but lethargic. Respiratory system: Bilateral lung sounds were coarse, with no dry or wet rales. Cardiovascular system: Regular rhythm, no pathological murmurs or pericardial friction rubs. Abdomen: Soft and flat, with mild epigastric tenderness on palpation, no rebound pain, negative Murphy’s sign, and no liver percussion pain. Bowel sounds were 3 beats/min. Neurological examination: Muscle strength was grade V in both upper limbs and grade IV in both lower limbs. Pathologic reflexes were negative. Electrocardiogram (ECG) showed sinus tachycardia (Figure 1A).

Laboratory findings included: White blood cell count: 11.33 × 10^9^/L (elevated). Creatine kinase: 297 U/L. Creatine kinase isoenzyme: 36 U/L. Blood glucose: 7.21 mmol/L. Coagulation, liver and renal function, electrolytes, and lipid profiles: Within normal limits. Preliminary diagnosis: Drug poisoning.

Initial treatment included: Cardiac monitoring and oxygen supplementation. Establishment of intravenous access for fluid resuscitation. Gastric lavage, activated charcoal administration, and catharsis to eliminate toxins. Protection of the gastric mucosa.

Clinical course: At 17:23, the patient suddenly lost consciousness, with shallow and weak respirations, blood pressure dropping to 65/44 mmHg, bilateral pupils 3 mm (sluggish light reflex), and SpO₂ of 40%. ECG indicated ventricular fibrillation (see Figure 1B). Immediate interventions included: Cardiopulmonary resuscitation (CPR). Intravenous injection of 1 mg of epinephrine. Asynchronous electrical defibrillation (360 J monophasic). Antiarrhythmic therapy with 1 mg of atropine, 300 mg of amiodarone. Transoral intubation and ventilator-assisted ventilation. Right femoral vein cannulation and continuous infusion of norepinephrine (2 μg/kg/min) to maintain blood pressure.

Despite a transient restoration of heart rhythm, the patient repeatedly experienced ventricular fibrillation at the 9th, 14th, 16th, 18th, and 22nd minutes, necessitating multiple defibrillations. During this period, lidocaine was administered. At 46 min, ECG showed ventricular tachycardia (Figure 1C), with blood pressure at 75/48 mmHg. At 63 min, the patient developed ventricular fibrillation, which rapidly progressed to ventricular arrest. Although pulsatile ventricular tachycardia was briefly restored, it could not be sustained, with episodes recurring every 1–2 min. By 120 min, the patient regained an autonomic rhythm, with ECG showing ventricular tachycardia (Figure 1D). The patient’s family refused blood purification therapy due to financial constraints.

Final status: Deep coma (Glasgow Coma Scale score: 3). Blood pressure: 90/40 mmHg (on norepinephrine infusion). Heart rate: 120 beats/min. Arterial blood gas: pH 6.57, PaCO_2_ 58.40 mmHg, PaO_2_ 175.00 mmHg, HCO_3_^−^5 mmol/L, BE-30 mmol/L, lactate 9.82 mmol/L. Electrolytes: Sodium 140 mmol/L, potassium 4.70 mmol/L, calcium 0.76 mmol/L. Ongoing treatment: Ventilator-assisted ventilation. Antiarrhythmic therapy with amiodarone. Continuous norepinephrine infusion for blood pressure support. Gastric tube administration of activated charcoal. Correction of electrolyte and acid–base imbalances. Anti-inflammatory therapy and albumin infusion.

**Figure 2:**
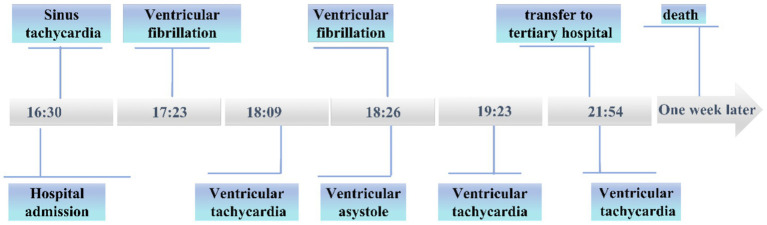
Flowchart of patient progression.

Outcome: At 21:54, the patient’s heart rate was 103 beats per minute, blood pressure was 95/45 mmHg, and the electrocardiogram (ECG) indicated ventricular tachycardia (with accompanying Figure 1E). The blood gas analysis showed: pH 7.15, PCO_2_ 40.40 mmHg, PaO_2_ 63 mmHg, HCO_3_^−^ 13.50 mmol/L, BE −15 mmol/L, and Lac 8.74 mmol/L. The electrolyte levels were: sodium 142 mmol/L, potassium 4 mmol/L, and calcium 0.9 mmol/L. After communicating with the patient’s family, the patient was transferred to a higher-level hospital for further treatment but succumbed to the condition 1 week later despite aggressive interventions. A detailed flow chart of the patient’s clinical progression is provided in Figure 2.

## 3 Materials and methods

### 3.1 Materials and reagents

Xiaozhong Jiuwei Powder (XZJWP) was purchased from Otaqi Pharmaceutical Co., Ltd., which is from the same manufacturer as the patient’s XZJWP. LC–MS grade methanol and formic acid were obtained from Merck (New Jersey, USA) and Fisher Scientific (New Jersey, USA), respectively. Deionized water was purchased from Watsons (Guangzhou, China), and analytical-grade methanol was supplied by Tianjin Xinbote Chemical Technology Co. All chemicals and reagents were of the highest purity available and used without further purification.

### 3.2 Sample preparation

Accurately weigh 10 mg of XZJWP and mix it with 500 μL of methanol. Sonicate the mixture at 40 kHz and 500 W ultrasonic power for 30 min, followed by centrifugation at 13,000 rpm for 10 min. Collect the supernatant for UHPLC-Q-Orbitrap-HRMS analysis, ensuring that the sample storage time does not exceed 24 h.

### 3.3 UHPLC-Q-Orbitrap-HRMS analysis method of XZJWP

The detailed chromatographic and mass spectrometry parameters, essential for reproducibility, are provided as follows. The chemical profiling of XZJWP was performed using a UHPLC-Q-Orbitrap-HRMS system consisting of a U3000 UHPLC coupled to a Q-Exactive mass spectrometer (Thermo Fisher Scientific). Chromatographic separation was achieved on a Thermo Hypersil Gold C18 column (100 mm × 2.1 mm, 1.9 μm) with a mobile phase of 0.1% formic acid in water (A) and methanol (B). The gradient program was as follows: 0–1 min, 10% B; 1–15 min, 10–100% B; 15–17 min, 100% B; 17–17.1 min, 100–10% B; 17.1–20 min, 10% B, at a constant flow rate of 0.3 mL/min and an injection volume of 10 μL. Mass spectrometric analysis utilized a Heated Electrospray Ionization (HESI) source with the heater and capillary temperatures set to 310 °C and 320 °C, respectively. Sheath and auxiliary gas pressures were 30 and 10 arb, with spray voltages of 3.0 kV (positive) and 2.8 kV (negative). Full MS scans (m/z 100–1,500) were acquired at a resolution of 70,000, with an AGC target of 3e6 and maximum injection time of 200 ms. Data-dependent MS/MS acquisition was performed on the top 10 ions at a resolution of 17,500 (AGC target 1e5, max IT 50 ms), using stepped normalized collision energies of 10, 28, and 35 eV.

## 4 Results and discussion

**Figure 3:**
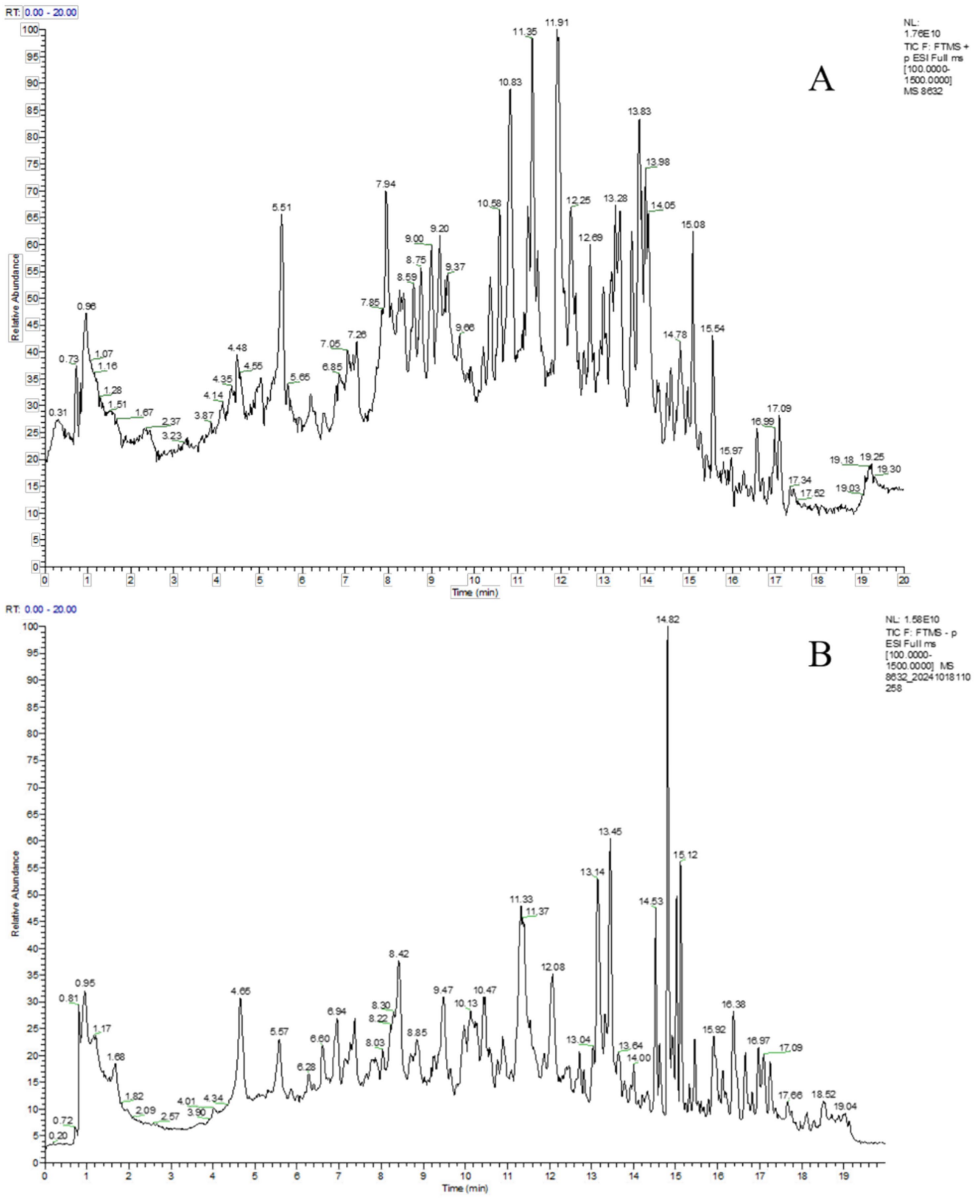
The total ion chromatograms of Xiaozhong Jiuwei San powder extract in positive **(A)** and negative **(B)** ion modes.

Chemical profiling of XZJWP was conducted using UHPLC-Q-Orbitrap-HRMS in both positive and negative ion modes. The acquired data were analyzed using Compound Discoverer software (v3.2, Thermo Fisher Scientific, CA, USA), which automatically searched online databases: ChemSpider, CHEBI, CHEMBL, Natural Products Database, Flavonoid databases, OTC databases, and the mzCloud database. A total of 292 components from all nine herbal ingredients were characterized based on database matching and fragmentation patterns. Figure 3 illustrates the total ion chromatogram of the XZJWP extract obtained from UHPLC-Q-Orbitrap-HRMS analysis in both ionization modes.

The identified compounds were classified into 24 structural categories, including 31 alkaloids, 52 flavonoids, 33 terpenoids, 36 fatty acids, 18 phenolic acids, 17 organic acids, 12 anthraquinones, 12 saccharides, 11 amino acids, 10 coumarins, 8 volatile oils, 10 amides, 5 benzaldehyde, 5 lactones, 4 steroid saponins, 4 polyphenols, 2 furfural, 1 naphthalene glycoside, 1 stilbene, 1 hydrocarbon, 1 aromatic amines, 1 protein, 1 lignin, 1 phenylpropanoid and others. Detailed information on retention times, molecular ions, mass errors, MS/MS fragmentation patterns, identified compounds, and classifications is provided in Supplementary Table 1.

**Figure 4:**
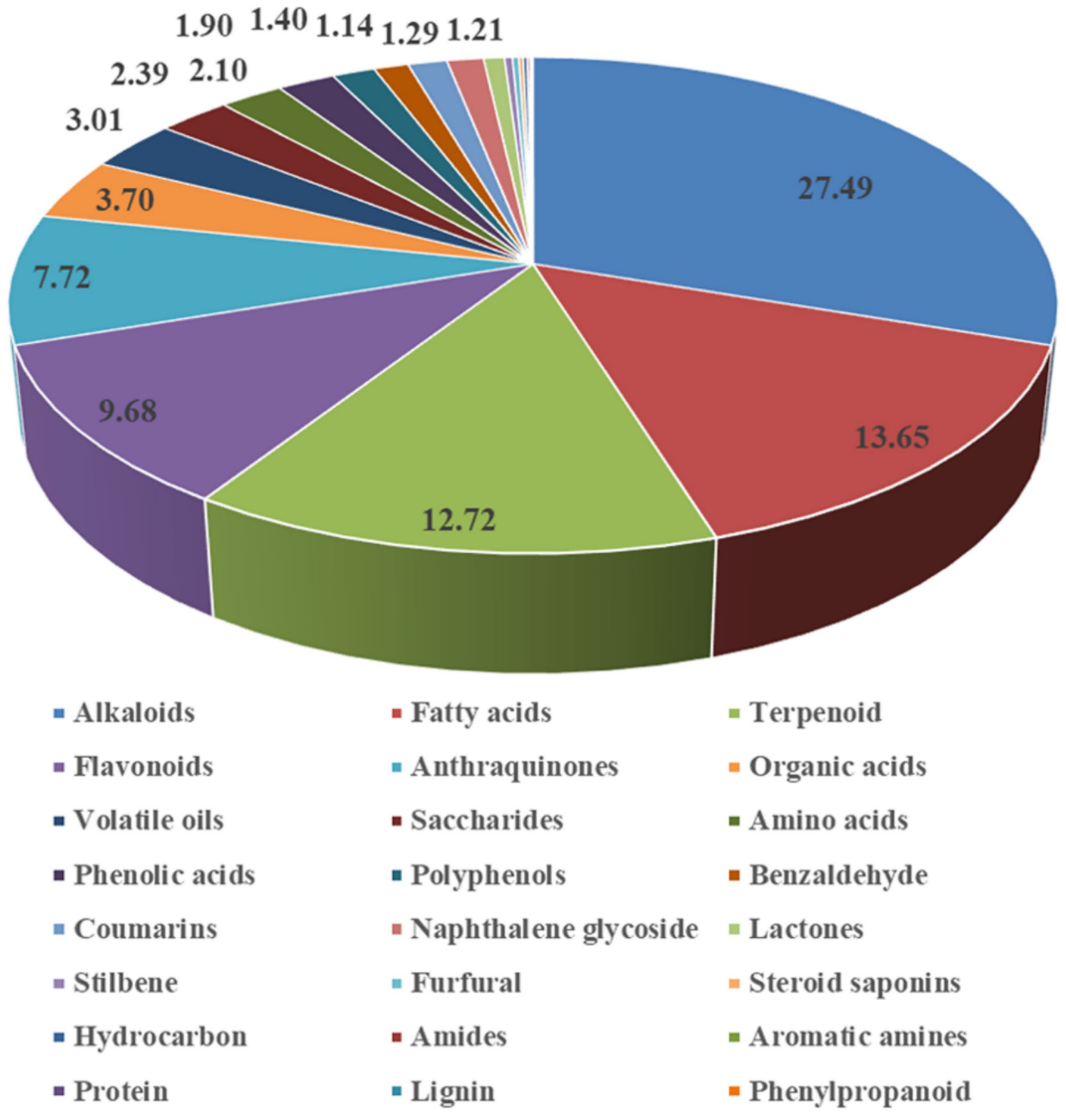
Percentage of substance detected for each chemical group classification in the Xiaozhong Jiuwei San powder extract.

A semi-quantitative analysis was performed by calculating the relative proportions of each compound based on peak area percentages (compound peak area/total peak area). The results revealed (Figure 4) that the most abundant compound classes in XZJWP extract were alkaloids (27.49%), fatty acids (13.65%), terpenoids (12.72%), flavonoids (9.68%), and anthraquinones (7.72%). The top ten compounds with the highest concentrations were fuziline (7.50%), benzoylmesaconine (6.55%), curcumenol (6.26%), curdione (5.62%), emodin (4.08%), linoleic acid (3.62%), mesaconitine (3.25%), epicatechin (3.25%), *β*-asarone (2.73%), and hypaconitine (2.70%).

**Figure 5:**
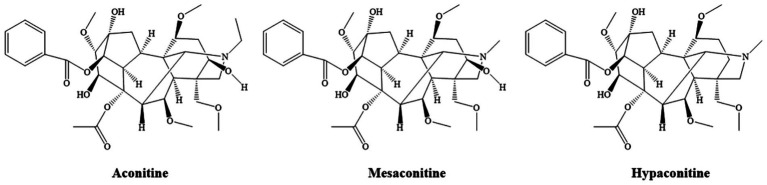
Chemical structure of the major diester-diterpenoid alkaloids.

The results showed that alkaloids were the most abundant chemical constituents in XZJWP, which is consistent with the presence of *Aconitum kusnezoffii* Rchb. in the formulation. Aconitine alkaloids, the bioactive components unique to the Aconitum species, are known for their analgesic, anti-inflammatory, antioxidant, and antitumor properties. However, their narrow therapeutic window poses significant risks, including cardiotoxicity, hepatotoxicity, muscle toxicity, and neurotoxicity (17–20). Notably, the diester-diterpenoid alkaloids (DDAs), such as aconitine, mesaconitine, and hypaconitine (Figure 5), are highly toxic and are typically reduced through processing methods before clinical use. In this study, these three DDAs were detected in high concentrations in the XZJWP extract.

Die-ester diterpenoid alkaloids (DDAs), such as aconitine, mesaconitine, and hypaconitine, are highly toxic components found in Aconitum species (e.g., such as *Aconiti Lateralis Radix*, *Aconiti Radix*, and *Aconiti Kusnezoffii Radix*). Their toxicity is primarily mediated by the interaction with voltage-gated sodium channels (Nav) in excitable tissues. DDAs bind to the open state of these channels, potently inhibiting their inactivation. This leads to a persistent Na^+^ influx, sustained membrane depolarization, and ultimately, a refractory state of the tissue. This pathological sustained depolarization promotes early afterdepolarizations (EADs) and delayed afterdepolarizations (DADs), which are the fundamental triggers for severe ventricular arrhythmias (21, 22). Beyond the sodium channel effects, DDAs disrupt intracellular Ca^2+^ homeostasis. Studies indicate that aconitine induces excessive Ca^2+^ influx in ventricular myocytes, impairing the Na^+^/Ca^2+^ exchanger and sarcoplasmic reticulum Ca^2+^-ATPase function, culminating in cytotoxic calcium overload (23). Furthermore, these alkaloids induce cardiomyocyte apoptosis and oxidative damage by directly targeting mitochondrial function, multiple ion channels, and connexin 43 (24–26). The presence of the diester moiety in their molecular structure is considered critical for this potent toxicity.

According to the literature (27), oral administration of 0.2–2 mg of aconitine can cause poisoning, while 3–5 mg can be fatal. Intramuscular injection of 0.2–0.3 mg can also lead to death, frequently resulting in malignant arrhythmias including ventricular tachycardia and fibrillation. In this case, the patient was aggressively managed with a combination of lidocaine (a class Ib antiarrhythmic that inhibits Na^+^ influx) and amiodarone, in addition to atropine to counteract vagal depression. Despite this conventional intensive care, the patient succumbed, starkly highlighting the therapeutic challenges in severe DDA-induced cardiotoxicity.

This fatal outcome underscores the limitations of standard antiarrhythmic therapy alone. A comparative analysis of the three most recent aconitine poisoning reports (28–30) clarifies the critical factors determining survival. Current evidence suggests that the cornerstone of managing severe aconitine poisoning involves immediate gastrointestinal decontamination (e.g., gastric lavage), vigorous fluid diuresis, and aggressive correction of electrolyte imbalances. When malignant arrhythmias occur, conventional antiarrhythmic drugs are first-line; however, the development of refractory ventricular fibrillation (VF) necessitates a paradigm shift. In such cases, extracorporeal support, particularly extracorporeal membrane oxygenation (ECMO), is increasingly regarded as a first-line antidotal intervention to sustain circulation and break the fatal cycle of electrical storm, thereby facilitating toxin clearance.

Our patient’s continued deterioration, despite receiving standard intensive care, can therefore be attributed to the progression of refractory VF in the absence of this pivotal advanced support. Tragically, due to financial constraints, life-sustaining interventions like ECMO or hemoperfusion could not be implemented. This case aligns with previous reports where fatal outcomes are often linked to the inability to reverse the profound cardiotoxic cascade (31), and it powerfully emphasizes that successful resuscitation from severe aconitine poisoning may hinge on the timely deployment of extracorporeal life support when conventional measures fail.

The chemical profiling of XZJWP using UHPLC-Q-Orbitrap-HRMS provided valuable insights into the composition of the formulation, revealing the presence of 292 components across 24 structural categories, with alkaloids being the most abundant class. While these compounds contribute to the therapeutic effects of XZJWP, the high concentrations of toxic DDAs emphasize the need for stringent quality control, proper processing methods, and precise dosing to mitigate the risks of toxicity. This case serves as a critical reminder of the potential dangers associated with the misuse of herbal medicines, particularly those containing aconitine alkaloids. It underscores the importance of educating healthcare providers and patients about the risks of DDA-containing formulations, ensuring they are aware of the narrow therapeutic window and potential for severe toxicity. Additionally, rigorous quality control measures must be implemented to guarantee the safety and efficacy of herbal products, including proper processing methods to reduce toxic components like DDAs. Further research is essential to better understand the pharmacokinetics, pharmacodynamics, and toxicology of DDAs and other bioactive compounds in XZJWP, which will inform safer clinical practices. Finally, standardized guidelines for the safe use of traditional herbal medicines must be developed, particularly for formulations containing potent and toxic compounds, to prevent adverse outcomes and ensure patient safety. This multifaceted approach is crucial for balancing the therapeutic benefits of herbal medicines with the need to minimize risks.

In conclusion, while XZJWP holds potential therapeutic value, its improper use can lead to severe and potentially fatal outcomes, as demonstrated in this case. A balanced approach that integrates traditional knowledge with modern scientific methods is essential to maximize the benefits and minimize the risks associated with such formulations. This case report and chemical profiling study contribute to the growing body of evidence on the safe and effective use of herbal medicines, emphasizing the need for caution and vigilance in their clinical application.

## Data Availability

The original contributions presented in the study are included in the article/Supplementary material, further inquiries can be directed to the corresponding authors.
