# Supplementary material for: A fatal intractable ventricular fibrillation triggered by improper use of Xiaozhong Jiuwei Powder: integrated LC-MS chemical profiling unveils the cardiotoxic culprits
Source: Front Med (Lausanne). 2025 Oct 27;12:1682854. doi: 10.3389/fmed.2025.1682854 (PMC12597811; doi:10.3389/fmed.2025.1682854)
Supplement: Supplementary file 1 [file Table_1.docx]

A Fatal Intractable Ventricular Fibrillation Triggered by Improper Use of Xiaozhong Jiuwei Powder: Integrated LC-MS Chemical Profiling Unveils the Cardiotoxic Culprits

Halidan Abudu ^a, b,^ ^†^, Zhaofei Yang ^c, †^, Lu Zhang ^c^, Dilidaer Sidike ^d^, Liqiong Guo ^a^, Haojun Fan ^a,*^ and Ayixiamuguli Wubuli ^b,*^

^a,#^*School of Disaster and Emergency Medicine, Tianjin University, Tianjin 300072, China*

^b,#^*The Key Laboratory of Xinjiang Endemic and Ethnic Diseases and Department of Biochemistry, Shihezi University School of Medicine, Shihezi 832002, China*

^c^*Emergency Department, People's Hospital of Changji Hui Autonomous Prefecture, Changji 831100, China*

^d^*Department of Oncology, The First Affiliated Hospital of Xinjiang Medical University, Urumqi 830054, China*

^*^Correspondence: haojunfan86@163.com; ayixiamugu18@mails.ucas.ac.cn

^†^These authors contributed equally to this work

^#^These affiliations (a and b) are co-primary and contributed equally to this work

Table S1. Identification of chemical components of XZJWP extract by UHPLC-Q-Exactive-Orbitrap MS

| No | t_R_(min) | Ion mode | Molecular ion | Molecular Formula | ppm Error | Identification | Classification | Percentage Peak Area |
| --- | --- | --- | --- | --- | --- | --- | --- | --- |
| 1 | 5.508 | [M+H] | 454.27939 | C24H39NO7 | -1.1228 | Fuziline | Alkaloids | 7.5012 |
| 2 | 7.943 | [M+H] | 590.29517 | C31H43NO10 | 1.011 | Benzoylmesaconine | Alkaloids | 6.5527 |
| 3 | 11.948 | [M+H] | 235.16888 | C15H22O2 | -1.5827 | Curcumenol | Sesquiterpenes | 6.2613 |
| 4 | 11.35 | [M+H] | 237.18468 | C15H24O2 | -0.9673 | Curdione | Terpenoid | 5.6210 |
| 5 | 13.163 | [M-H] | 269.04562 | C15H10O5 | 4.3632 | Emodin | Anthraquinones | 4.0774 |
| 6 | 14.812 | [M-H] | 279.23288 | C18H32O2 | 3.6633 | Linoleic acid | Fatty acids | 3.6213 |
| 7 | 8.993 | [M+H] | 632.30573 | C33H45NO11 | -0.4739 | Mesaconitine | Alkaloids | 3.2505 |
| 8 | 4.648 | [M-H] | 289.0719 | C15H14O6 | 4.0605 | Epicatechin | Flavonoids | 3.2463 |
| 9 | 10.805 | [M+H] | 209.1171 | C12H16O3 | -0.8165 | β-Asarone | Volatile oil | 2.7304 |
| 10 | 9.194 | [M+H] | 616.31097 | C33H45NO10 | 1.0267 | Hypaconitine | Alkaloids | 2.7042 |
| 11 | 1.669 | [M-H] | 169.01347 | C7H6O5 | 1.9156 | Gallic acid | Organic acids | 2.2553 |
| 12 | 15.124 | [M-H] | 281.24857 | C18H34O2 | 3.7649 | Oleic Acid | Fatty acids | 2.0587 |
| 13 | 11.24 | [M+H] | 247.13268 | C15H18O3 | -0.79228 | Santonin | Terpenoids | 1.9758 |
| 14 | 13.442 | [M-H] | 295.22784 | C18H32O3 | 3.6342 | 9-hydroxy-10,12-octadecadienoic acid | Fatty acids | 1.5529 |
| 15 | 10.256 | [M-H] | 431.09839 | C21H20O10 | 2.6584 | Apigenin-7-O-glucoside | Flavonoids | 1.5185 |
| 16 | 12.084 | [M-H] | 283.0249 | C15H8O6 | 3.9816 | Rhein | Anthraquinones | 1.5070 |
| 17 | 12.694 | [M+H] | 217.15849 | C15H20O | -0.91541 | ar-Turmerone | Flavonoids | 1.4682 |
| 18 | 15.025 | [M-H] | 255.23282 | C16H32O2 | 3.7686 | Palmitic Acid | Fatty acids | 1.3452 |
| 19 | 13.393 | [M+H] | 279.23154 | C18H30O2 | -1.14552 | α-Linolenic acid | Fatty acids | 1.3211 |
| 20 | 11.471 | [M+H] | 286.1434 | C17H19NO3 | -1.0724 | Piperine | Alkaloids | 1.2693 |
| 21 | 9.465 | [M-H] | 407.13486 | C20H24O9 | 2.9437 | Tinnevellin glucoside | Naphthalene glycoside | 1.2073 |
| 22 | 8.587 | [M+H] | 233.15332 | C15H20O2 | -1.2272 | Costunolide | Sesquiterpene lactones | 1.2022 |
| 23 | 11.916 | [M+H] | 231.13776 | C15H18O2 | -0.8654 | Dehydrocostus lactone | Sesquiterpenoids | 1.0762 |
| 24 | 6.609 | [M-H] | 441.08282 | C22H18O10 | 2.7242 | epicatechin gallate | Polyphenols | 0.9486 |
| 25 | 0.94 | [M+NH4] | 116.07059 | C5H9NO2 | -0.1545 | 2-Pyrrolidinecarboxylic acid | Alkaloids | 0.9472 |
| 26 | 5.65 | [M+H+MeOH] | 438.28461 | C24H39NO6 | -0.9297 | Neoline/Bullatine B | Alkaloids | 0.9004 |
| 27 | 1.545 | [M+H] | 132.1019 | C6H13NO2 | -0.0532 | L-Leucine | Alkaloids | 0.8808 |
| 28 | 12.999 | [M+H] | 219.17419 | C15H22O | -0.67323 | Germacrone | Sesquiterpenoids | 0.8558 |
| 29 | 13.465 | [M-H] | 253.05055 | C15H10O4 | 4.0193 | rubiadin | Anthraquinones | 0.7265 |
| 30 | 16.973 | [M-H] | 293.17932 | C14H30O4S | 3.093 | Myristyl sulfate | others | 0.7192 |
| 31 | 1.833 | [M-H] | 387.11465 | C12H22O11 | 3.4549 | Lactose | Saccharides | 0.7037 |
| 32 | 7.947 | [M+H] | 197.08063 | C10H12O4 | -1.05514 | 2,4,5-Trimethoxybenzaldehyde | others | 0.6771 |
| 33 | 4.118 | [M+H] | 188.07048 | C11H9NO2 | -0.90673 | trans-3-Indoleacrylic acid | Alkaloids | 0.6395 |
| 34 | 9.373 | [M+H] | 409.14902 | C20H24O9 | -0.8614 | Nodakenin | Coumarins | 0.6227 |
| 35 | 8.072 | [M+H] | 249.14824 | C15H20O3 | -1.0702 | Atractylenolide III | Lactones | 0.5886 |
| 36 | 10.936 | [M-H] | 285.04059 | C15H10O6 | 4.0797 | Luteolin | Flavonoids | 0.5538 |
| 37 | 1.001 | [M-H] | 133.01318 | C4H6O5 | 0.2544 | Malic acid | Phenolic acids | 0.5514 |
| 38 | 2.452 | [M+NH4] | 166.08621 | C9H11NO2 | 0.01671 | L-Phenylalanine | Amino acids | 0.5204 |
| 39 | 6.486 | [M+H] | 443.09695 | C22H18O10 | -0.99503 | Epicatechin gallate | Phenolic acids | 0.5198 |
| 40 | 14.522 | [M-H] | 277.21713 | C18H30O2 | 3.7803 | α-Eleostearic acid | Fatty acids | 0.5166 |
| 41 | 9.295 | [M+H] | 646.32117 | C34H47NO11 | 0.59266 | Aconitine | Alkaloids | 0.5082 |
| 42 | 0.758 | [M+H] | 175.11896 | C6H14N4O2 | 0.03009 | L-Arginine | Amino acids | 0.4972 |
| 43 | 1.299 | [M+FA-H] | 387.11462 | C12H22O11 | 3.3761 | Sucrose | Saccharides | 0.4811 |
| 44 | 7.264 | [M+FA-H] | 355.10367 | C15H18O7 | 3.774 | 1-O-trans-cinnamoyl-beta-D-glucopyranose | Saccharides | 0.4742 |
| 45 | 0.966 | [M-H] | 191.0191 | C6H8O7 | 2.4866 | Citric acid | Organic acids | 0.4687 |
| 46 | 7.175 | [M+H-H2O] | 131.0491 | C9H8O2 | -0.29475 | p-Coumaraldehyde | Coumarins | 0.4226 |
| 47 | 8.034 | [M+FA-H] | 463.12491 | C21H22O9 | 3.0673 | Liquiritin | Flavonoids | 0.4080 |
| 48 | 10.791 | [M+H] | 285.07544 | C16H12O5 | -1.08931 | Glycitein | Amino acids | 0.3809 |
| 49 | 1.214 | [M-H] | 128.03426 | C5H7NO3 | 0.2843 | 4-Oxoproline | Alkaloids | 0.3792 |
| 50 | 11.402 | [M-H] | 269.04556 | C15H10O5 | 4.1364 | Aloeemodin | Anthraquinones | 0.3743 |
| 51 | 10.138 | [M-H] | 415.10367 | C21H20O9 | 3.155 | Chrysophanol 8-O-β-D-glucoside | Anthraquinones | 0.3665 |
| 52 | 9.481 | [2M+Na] | 257.08057 | C9H10O | -1.0463 | 2,4-Dimethylbenzaldehyde | Benzaldehyde | 0.3658 |
| 53 | 10.705 | [M+H] | 221.18982 | C15H24O | -0.78039 | Caryophyllene oxide | Terpenoids | 0.3633 |
| 54 | 5.708 | [M+H] | 422.28964 | C24H39NO5 | 4.43113 | Talatisamine | Alkaloids | 0.3586 |
| 55 | 0.805 | [M+H] | 104.10691 | C5H13NO | -0.76762 | Choline | Alkaloids | 0.3576 |
| 56 | 11.401 | [M-H] | 329.2334 | C18H34O5 | 3.4864 | (15Z)-9,12,13-Trihydroxy-15-octadecenoic acid | Fatty acids | 0.3556 |
| 57 | 15.087 | [M+H] | 441.37198 | C30H48O2 | -1.65065 | Roburic acid | Terpenoids | 0.3528 |
| 58 | 15.468 | [M-H] | 283.26422 | C18H36O2 | 3.7574 | Stearic acid | Fatty acids | 0.3461 |
| 59 | 8.78 | [M-H] | 187.09694 | C9H16O4 | 2.3431 | AZELAIC ACID | Fatty acids | 0.3422 |
| 60 | 7.967 | [M+H] | 419.13303 | C21H22O9 | -1.50923 | Liquiritin | Flavonoids | 0.3417 |
| 61 | 13.329 | [M-H] | 293.21237 | C18H32O4 | 4.265 | 9-HpODE | Fatty acids | 0.3390 |
| 62 | 16.974 | [M-H] | 367.35812 | C24H48O2 | 3.1526 | Lignoceric acid | Fatty acids | 0.3376 |
| 63 | 13.249 | [M+H] | 219.17416 | C15H22O | -1.26328 | Muurolladie-3-One | Others | 0.3355 |
| 64 | 4.458 | [M+H] | 378.26331 | C22H35NO4 | -1.69314 | Karakoline | Alkaloids | 0.3268 |
| 65 | 1.234 | [M-H] | 173.00841 | C6H6O6 | 1.984 | Aconitic Acid | Organic acids | 0.3050 |
| 66 | 11.809 | [M+H] | 233.1535 | C15H20O2 | -0.37638 | Isoalantolactone | Terpenoids | 0.2936 |
| 67 | 7.261 | [M+H] | 167.07019 | C9H10O3 | -0.4806 | o-Veratraldehyde | Others | 0.2887 |
| 68 | 0.943 | [M+H] | 118.08617 | C5H11NO2 | -0.67617 | L-Valine | Amino acids | 0.2820 |
| 69 | 10.132 | [M+H] | 271.05978 | C15H10O5 | -1.16577 | Apigenin | Flavonoids | 0.2755 |
| 70 | 1.013 | [M-H] | 225.06122 | C6H12O6 | 3.2868 | Galactose | Saccharides | 0.2633 |
| 71 | 3.631 | [M+H] | 143.03384 | C6H6O4 | -0.2882 | Kojic acid | Others | 0.2601 |
| 72 | 6.276 | [M+FA-H] | 435.12979 | C20H22O8 | -1.5492 | trans-piceid | stilbene | 0.2598 |
| 73 | 8.262 | [M+H] | 395.13333 | C19H22O9 | -0.8286 | aloesin | Anthraquinones | 0.2495 |
| 74 | 4.566 | [M+H] | 360.2529 | C22H33NO3 | -1.1693 | Napelline | Alkaloids | 0.2478 |
| 75 | 1.603 | [M-H] | 129.01828 | C5H6O4 | 0.3479 | Citraconic acid | Fatty acids | 0.2228 |
| 76 | 7.167 | [M+H] | 312.12277 | C18H19NO5 | -1.2315 | N-Feruloyloctopamine | Alkaloids | 0.2103 |
| 77 | 3.545 | [M+H] | 127.03893 | C6H6O3 | -0.3577 | 5-Hydroxymethylfurfural | furfural | 0.2093 |
| 78 | 16.408 | [M-H] | 339.32684 | C22H44O2 | 3.201 | Docosanoic acid | Fatty acids | 0.2089 |
| 79 | 11.399 | [M-H] | 337.10831 | C20H18O5 | 3.4864 | Demethoxycurcumin | Phenolic acids | 0.2060 |
| 80 | 1.672 | [M-H] | 125.02335 | C6H6O3 | 0.3252 | Pyrogallol | Polyphenols | 0.2041 |
| 81 | 13.928 | [M+H] | 285.07529 | C16H12O5 | -1.6246 | Emodin-3-methyl ether/Physcion | Anthraquinones | 0.1986 |
| 82 | 10.887 | [M-H] | 283.06122 | C16H12O5 | 3.9508 | Glycitein | Flavonoids | 0.1874 |
| 83 | 6.161 | [M+H] | 391.13846 | C20H22O8 | -0.7308 | Polydatin | Anthraquinones | 0.1788 |
| 84 | 10.835 | [M-H] | 327.21783 | C18H32O5 | 3.771 | Corchorifatty acid F | Fatty acids | 0.1712 |
| 85 | 9.065 | [M+H] | 231.13776 | C15H18O2 | -0.7994 | Atractylenolide I | Terpenoids | 0.1689 |
| 86 | 11.239 | [M+H] | 139.03886 | C7H6O3 | -0.7658 | Protocatechualdehyde | Polyphenols | 0.1684 |
| 87 | 4.315 | [M-H] | 137.02342 | C7H6O3 | 0.7421 | Salicylic acid | Organic acids | 0.1564 |
| 88 | 16.487 | [M-H] | 365.34253 | C24H46O2 | 2.988 | Nervonic acid | Fatty acids | 0.1454 |
| 89 | 9.837 | [M+ACN+H] | 369.13284 | C21H20O6 | -1.1427 | Curcumin | Phenolic acids | 0.1404 |
| 90 | 1.276 | [M+H] | 182.08121 | C9H11NO3 | 0.4574 | L-Tyrosine | Amino acids | 0.1380 |
| 91 | 13.633 | [M+H] | 205.19487 | C15H24 | -1.0105 | HUMULENE (alpha) | Sesquiterpenoids | 0.1374 |
| 92 | 10.891 | [M+H] | 165.12727 | C11H16O | -0.7155 | Jasmone | hydrocarbon | 0.1344 |
| 93 | 13.239 | [M-H] | 315.25403 | C17H34O2 | 3.3061 | Methyl hexadecanoate | Fatty acids | 0.1343 |
| 94 | 0.919 | [M+H] | 176.10295 | C6H13N3O3 | -0.0952 | L-citrulline | Amino acids | 0.1298 |
| 95 | 15.917 | [M-H] | 311.29568 | C20H40O2 | 3.6502 | Arachidic acid | Fatty acids | 0.1288 |
| 96 | 11.4 | [M-H+HAc] | 367.11868 | C19H16O4 | 2.9094 | Bisdemethoxycurcumin | Phenolic acids | 0.1282 |
| 97 | 10.887 | [M+FA-H] | 491.11972 | C22H22O10 | -2.222 | Biochanin-7-O-glucoside | Flavonoids | 0.1223 |
| 98 | 0.938 | [M+H] | 136.06175 | C5H5N5 | -0.143 | Adenine | others | 0.1211 |
| 99 | 1.067 | [M-H] | 181.07103 | C6H14O6 | 2.0162 | Mannitol | Saccharides | 0.1204 |
| 100 | 3.863 | [M+H] | 579.14899 | C30H26O12 | -1.2359 | Procyanidin B1 | Flavonoids | 0.1163 |
| 101 | 12.73 | [M-H] | 313.23856 | C18H34O4 | 3.992 | 12(13)-DiHOME | others | 0.1152 |
| 102 | 7.304 | [M-H] | 147.04417 | C9H8O2 | 0.8037 | trans-Cinnamic acid | Phenolic acids | 0.1113 |
| 103 | 11.439 | [M+H] | 1031.5415 | C51H82O21 | -0.6125 | Pseudoprotodioscin | others | 0.1098 |
| 104 | 12.304 | [M+H] | 489.35682 | C30H48O5 | -1.1198 | Asiatic acid | Terpenoids | 0.1085 |
| 105 | 11.89 | [M+H] | 439.21088 | C26H30O6 | -1.2457 | Kurarinone | Flavonoids | 0.1077 |
| 106 | 10.032 | [M+H] | 255.06502 | C15H10O4 | -0.6572 | Daidzein | Flavonoids | 0.1018 |
| 107 | 12.691 | [M-H+HAc] | 351.2178 | C18H28O3 | 3.4264 | 12-oxo Phytodienoic Acid | others | 0.0965 |
| 108 | 6.985 | [M+H] | 191.10649 | C12H14O2 | -0.8542 | Ligustilide | Lactones | 0.0964 |
| 109 | 6.087 | [M+NH4] | 153.05452 | C8H8O3 | -0.66 | 2-Hydroxy-4-methoxybenzaldehyde | Volatile oil | 0.0952 |
| 110 | 15.997 | [M-H] | 337.31143 | C22H42O2 | 3.9277 | Erucic acid | Fatty acids | 0.0918 |
| 111 | 3.215 | [M-H] | 153.01849 | C7H6O4 | 1.6894 | PYROCATECHUIC ACID | Organic acid | 0.0880 |
| 112 | 7.68 | [M-H] | 477.06769 | C21H18O13 | 2.7052 | quercetin 3-O-glucuronide | Flavonoids | 0.0876 |
| 113 | 1.068 | [2M+NH4] | 262.12842 | C6H6N2O | -0.3656 | NIACINAMIDE | Alkaloids | 0.0864 |
| 114 | 1.079 | [M+H] | 124.03929 | C6H5NO2 | -0.1096 | NIACIN | Alkaloids | 0.0861 |
| 115 | 8.131 | [M+H] | 207.10136 | C12H16O4 | -0.9981 | Senkyunolide H | Phthalides | 0.0833 |
| 116 | 10.768 | [M+H] | 193.12222 | C12H16O2 | -0.6689 | Senkyunolide A | Phthalides | 0.0825 |
| 117 | 6.16 | [M+H] | 229.08588 | C14H12O3 | -0.1935 | Resveratrol | Polyphenols | 0.0823 |
| 118 | 15.555 | [M-H] | 309.28012 | C20H38O2 | 4.2484 | 11(Z)-Eicosenoic acid | Fatty acids | 0.0822 |
| 119 | 1.405 | [M-H] | 117.01823 | C4H6O4 | -0.0077 | Methylmalonic acid | Organic acids | 0.0811 |
| 120 | 7.203 | [M+H] | 271.05988 | C15H10O5 | -0.828 | Baicalein | Flavonoids | 0.0776 |
| 121 | 17.307 | [M-H] | 381.37384 | C25H50O2 | 3.131 | Pentacosanoic acid | Fatty acids | 0.0774 |
| 122 | 12.005 | [2M+Na] | 233.15343 | C15H20O2 | -0.769 | Atractylenolide II | Sesquiterpenoids | 0.0759 |
| 123 | 0.924 | [M-H] | 195.05038 | C6H12O7 | 2.3347 | Gluconic acid | Saccharides | 0.0757 |
| 124 | 2.981 | [M-H] | 255.05101 | C11H12O7 | 4.2384 | Piscidic Acid | Alkaloids | 0.0705 |
| 125 | 13.524 | [M+H] | 469.33072 | C30H44O4 | -1.1023 | Glabrolide | Terpenoids | 0.0691 |
| 126 | 6.687 | [M-H] | 163.03922 | C9H8O3 | 1.4995 | trans-4-Coumaric acid | Coumarins | 0.0690 |
| 127 | 1.301 | [M+FA-H] | 549.16748 | C18H32O16 | 1.1849 | Melezitose | Saccharides | 0.0688 |
| 128 | 13.993 | [M-H] | 285.0405 | C15H10O6 | 4.0797 | Kaempferol | Flavonoids | 0.0679 |
| 129 | 1.843 | [M-H] | 549.16754 | C18H32O16 | 2.5513 | Raffinose | Saccharides | 0.0647 |
| 130 | 12.583 | [M+H] | 293.21075 | C18H28O3 | -1.2512 | 12-Oxo phytodienoic acid | Others | 0.0646 |
| 131 | 14.661 | [M-H] | 253.21721 | C16H30O2 | 3.9578 | Palmitoleic acid | Fatty acids | 0.0640 |
| 132 | 9.379 | [M+H] | 273.07538 | C15H12O5 | -1.3607 | Naringenin | Flavonoids | 0.0634 |
| 133 | 11.541 | [M+H] | 231.13792 | C15H18O2 | -0.1392 | Lindenenol | Sesquiterpenoids | 0.0632 |
| 134 | 7.548 | [M+H-NH3] | 479.08167 | C21H18O13 | -0.7346 | Quercetin 3-O-β-D-Glucuronide | Flavonoids | 0.0620 |
| 135 | 6.79 | [M-H] | 206.08176 | C11H13NO3 | 3.1437 | N-acetylphenylalanine | Amino acids | 0.0620 |
| 136 | 14.01 | [M+H-H2O] | 455.3512 | C30H48O4 | -1.7031 | Maslinic acid | Terpenoids | 0.0594 |
| 137 | 12.585 | [M+H] | 487.34134 | C30H46O5 | -0.8852 | Quillaic acid | Terpenoids | 0.0586 |
| 138 | 14.534 | [M-H] | 487.34277 | C30H48O5 | 1.9954 | Asiatic acid | Terpenoids | 0.0576 |
| 139 | 9.38 | [M+ACN+H] | 229.08574 | C14H12O3 | -0.793 | xanthyletine | Coumarins | 0.0575 |
| 140 | 10.642 | [M+H] | 219.17409 | C15H22O | -0.4644 | α-Cyperone | Volatile oil | 0.0574 |
| 141 | 14.299 | [M+H] | 324.28922 | C20H37NO2 | -1.5136 | Linoleoyl ethanolamide | Amino acids | 0.0566 |
| 142 | 11.946 | [M+H] | 149.09595 | C10H12O | -1.1155 | ANETHOLE | Volatile oil | 0.0555 |
| 143 | 9.8 | [M-H] | 201.11269 | C10H18O4 | 2.6621 | 3-tert-Butyladipic acid | Organic acids | 0.0553 |
| 144 | 10.723 | [M+H] | 741.44025 | C39H64O13 | -2.3222 | Timosaponin A-III | Steroid saponins | 0.0548 |
| 145 | 5.541 | [M-H] | 121.02841 | C7H6O2 | 0.0489 | Benzoic acid | Organic acids | 0.0543 |
| 146 | 13.805 | [M+H] | 111.04392 | C6H6O2 | -2.3023 | Hydroquinone | Others | 0.0530 |
| 147 | 14.184 | [M+H-NH3] | 303.23145 | C20H30O2 | -1.3568 | Abietic Acid | Terpenoids | 0.0523 |
| 148 | 8.604 | [M+H] | 289.07028 | C15H12O6 | -1.7459 | Eriodictyol | Flavonoids | 0.0511 |
| 149 | 11.72 | [M+H] | 285.07541 | C16H12O5 | -1.1964 | Acacetin | Flavonoids | 0.0509 |
| 150 | 7.582 | [M+H+MeOH] | 463.12308 | C22H23O11 | -0.8864 | Peonidin-3-O-glucoside | Flavonoids | 0.0505 |
| 151 | 16.915 | [M+H] | 122.09637 | C8H11N | -0.7963 | N,N-Dimethylaniline | Aromatic amines | 0.0499 |
| 152 | 7.727 | [M-H] | 229.10779 | C11H18O5 | 3.2211 | 2-(6-Hydroxyhexyl)-3-methylenesuccinic acid | Organic acids | 0.0496 |
| 153 | 15.785 | [M-H] | 455.35318 | C30H48O3 | 2.6532 | Ursolic acid | Terpenoids | 0.0491 |
| 154 | 14.694 | [M+H] | 282.27884 | C18H35NO | -1.0705 | Oleamide | Amides | 0.0482 |
| 155 | 9.399 | [M-H] | 263.12891 | C15H20O4 | 4.1431 | Abscisic acid | Sesquiterpenoids | 0.0482 |
| 156 | 12.878 | [M+H] | 279.15887 | C16H22O4 | -0.7565 | Dibutyl phthalate | Organic acids | 0.0477 |
| 157 | 5.098 | [M+H] | 339.10706 | C16H18O8 | -0.9651 | gerberinside | Flavonoids | 0.0473 |
| 158 | 14.757 | [M+H] | 455.35132 | C30H46O3 | -1.435 | Oleanonic acid | Terpenoids | 0.0459 |
| 159 | 9.855 | [M+H] | 255.0649 | C15H10O4 | -1.1358 | Chrysin | Flavonoids | 0.0457 |
| 160 | 7.088 | [M-H] | 449.10928 | C21H22O11 | 3.2189 | Eriodictyol-7-O-glucoside | Flavonoids | 0.0456 |
| 161 | 9.347 | [M+H] | 595.14417 | C30H26O13 | -0.9649 | Kaempferol-3-O-glucoside-6''-p-coumaroyl | Flavonoids | 0.0452 |
| 162 | 8.203 | [M+H] | 463.08673 | C21H18O12 | -0.802 | Kaempferol 3-glucuronide | Flavonoids | 0.0450 |
| 163 | 5.665 | [M-H] | 179.03429 | C9H8O4 | 2.2414 | trans-Caffeic acid | Phenolic acids | 0.0442 |
| 164 | 15.78 | [M+H] | 349.2709 | C23H34O2 | -1.1736 | Docosahexaenoic acid methyl ester | Fatty acids | 0.0439 |
| 165 | 14.128 | [M+H] | 219.1741 | C15H22O | -1.2998 | Nootkatone | Sesquiterpenoids | 0.0437 |
| 166 | 7.648 | [M+H] | 467.0816 | C20H20O14 | -0.8841 | 1,6-Bis-O-(3,4,5-trihydroxybenzoyl)hexopyranose | Saccharides | 0.0430 |
| 167 | 14.477 | [M+H] | 355.28391 | C21H38O4 | -1.0728 | monolinolein | Protein | 0.0423 |
| 168 | 4.537 | [M+H] | 189.13843 | C12H16N2 | -1.0436 | N,N-Dimethyltryptamine | Alkaloids | 0.0401 |
| 169 | 1.318 | [M-H+HAc] | 711.22009 | C24H42O21 | 2.0166 | Nystose | Saccharides | 0.0398 |
| 170 | 14.666 | [M-H] | 471.34808 | C30H48O4 | 2.1512 | Echinocystic acid | Terpenoids | 0.0392 |
| 171 | 5.373 | [M+H] | 293.02881 | C13H8O8 | -1.0017 | Brevifolincarboxylic acid | Phenolic acids | 0.0391 |
| 172 | 9.21 | [M-H] | 301.03543 | C15H10O7 | 3.8263 | Quercetin | Flavonoids | 0.0384 |
| 173 | 15.244 | [M-H] | 307.2644 | C20H36O2 | 4.0598 | 11(Z),14(Z)-Eicosadienoic acid | Phenolic acids | 0.0380 |
| 174 | 8.653 | [M-H] | 447.09656 | C21H20O11 | -3.3623 | Kaempferol-3-O-glucoside | Flavonoids | 0.0377 |
| 175 | 11.297 | [M+H] | 259.09619 | C15H14O4 | -1.1349 | Luvangetin | Coumarins | 0.0373 |
| 176 | 8.216 | [M-H] | 185.08124 | C9H14O4 | 2.2566 | 1-(Carboxymethyl)  cyclohexanecarboxylic acid | Organic acids | 0.0366 |
| 177 | 4.493 | [M+H] | 123.04367 | C7H6O2 | -3.1319 | p-Hydroxybenzaldehyde | Volatile oil | 0.0366 |
| 178 | 6.357 | [M-H] | 951.07404 | C41H28O27 | 0.6465 | Geraniin | Flavonoids | 0.0365 |
| 179 | 12.891 | [M+FA-H] | 1093.50671 | C50H80O23 | 0.0932 | Terrestrosin D | Steroid saponins | 0.0363 |
| 180 | 7.532 | [M+H] | 259.09634 | C15H14O4 | -0.546 | ONONETIN | Flavonoids | 0.0356 |
| 181 | 7.402 | [M+H] | 179.0338 | C9H6O4 | -0.4859 | Esculetin | Coumarins | 0.0353 |
| 182 | 14.43 | [M+H] | 471.3468 | C30H46O4 | -0.0501 | 18 β-Glycyrrhetintic Acid | Terpenoids | 0.0347 |
| 183 | 7.172 | [M-H] | 193.05 | C10H10O4 | 2.4231 | trans-Ferulic acid | Phenolic acids | 0.0343 |
| 184 | 0.99 | [M-H] | 151.06023 | C5H12O5 | 0.8375 | ARABITOL(D) | Saccharides | 0.0340 |
| 185 | 9.886 | [M-H] | 431.09851 | C21H20O10 | 2.8707 | Apigenin-8-C-glucoside | Flavonoids | 0.0335 |
| 186 | 7.518 | [M-H] | 173.08118 | C8H14O4 | 1.9723 | Suberic acid | Organic acids | 0.0330 |
| 187 | 7.29 | [M+H] | 463.12338 | C22H22O11 | -0.2274 | Peonidin-3-O-beta-galactoside | Flavonoids | 0.0316 |
| 188 | 8.469 | [M-H] | 312.12436 | C18H19NO4 | 4.146 | feruloyltyramine | Alkaloids | 0.0309 |
| 189 | 7.659 | [M+H] | 229.08585 | C14H12O3 | -0.3267 | cis-resveratrol | Others | 0.0308 |
| 190 | 6.053 | [M-H] | 154.01367 | C6H5NO4 | 1.3182 | 4-Nitrocatechol | Alkaloids | 0.0285 |
| 191 | 7.041 | [M+H] | 195.06499 | C10H10O4 | -1.0158 | Ferulic acid | Phenolic acids | 0.0284 |
| 192 | 11.705 | [M+FA-H] | 1093.54272 | C51H84O22 | 1.7787 | Protodioscin | Steroid saponins | 0.0276 |
| 193 | 6.353 | [M-H] | 172.09712 | C8H15NO3 | 1.757 | 2-(Acetylamino)hexanoic acid | Alkaloids | 0.0264 |
| 194 | 14.782 | [M+Na] | 379.28143 | C21H40O4 | -1.1806 | Monoolein | Fatty acids | 0.0256 |
| 195 | 5.397 | [M-H] | 635.08911 | C27H24O18 | 1.9229 | 1,3,6-tri-O-galloylglucose | Saccharides | 0.0237 |
| 196 | 1.079 | [M+H] | 110.05999 | C6H7NO | -0.1096 | 4-Aminophenol | Alkaloids | 0.0234 |
| 197 | 11.971 | [M-H] | 437.19708 | C26H30O6 | 2.7847 | Kurarinone | Flavonoids | 0.0226 |
| 198 | 10.286 | [M+H] | 287.09113 | C16H14O5 | -0.9654 | Sakuranetin | Flavonoids | 0.0226 |
| 199 | 6.215 | [M-H] | 151.03908 | C8H8O3 | 0.7094 | Vanillin | Others | 0.0225 |
| 200 | 2.544 | [M-H] | 164.07086 | C9H11NO2 | 2.2538 | L-Phenylalanine | Amino acids | 0.0223 |
| 201 | 14.749 | [M-H] | 303.23306 | C20H32O2 | 3.9772 | Arachidonic acid | Fatty acids | 0.0222 |
| 202 | 6.738 | [M+H] | 165.0546 | C9H8O3 | -1.0444 | trans-4-Coumaric acid | Coumarins | 0.0218 |
| 203 | 13.743 | [M+H] | 417.33569 | C27H44O3 | -2.0907 | Sarsasapogenin | Steroid saponins | 0.0217 |
| 204 | 9.503 | [M-H] | 593.15179 | C27H30O15 | 2.8626 | Kaempferol-3-O-rutinoside | Flavonoids | 0.0213 |
| 205 | 4.91 | [M-H] | 577.13525 | C30H26O12 | 2.0816 | Procyanidin B2 | Flavonoids | 0.0210 |
| 206 | 4.607 | [M+H] | 175.12276 | C11H14N2 | -1.2454 | N-Methyltryptamine | Alkaloids | 0.0209 |
| 207 | 14.588 | [M+H] | 209.11691 | C12H16O3 | -1.4732 | α-Asarone | Others | 0.0206 |
| 208 | 4.078 | [M-H] | 145.04968 | C6H10O4 | 1.0158 | Adipic acid | Fatty acids | 0.0198 |
| 209 | 13.675 | [M-H] | 243.1964 | C14H28O3 | 3.5512 | (R)-3-Hydroxy myristic acid | Fatty acids | 0.0196 |
| 210 | 6.911 | [M-H] | 303.05106 | C15H12O7 | 3.7181 | Taxifolin | Flavonoids | 0.0186 |
| 211 | 11.242 | [M-H] | 249.14946 | C15H22O3 | 3.7681 | Nardosinone | Sesquiterpenoids | 0.0186 |
| 212 | 2.951 | [M-H] | 131.03397 | C5H8O4 | 0.7335 | Methylsuccinic acid | Organic acids | 0.0183 |
| 213 | 8.164 | [M+H] | 447.09167 | C21H18O11 | -1.1473 | apigenin-7-O-glucuronide | Flavonoids | 0.0180 |
| 214 | 10.543 | [M-H] | 347.18637 | C20H28O5 | 3.0832 | Ingenol | Terpenoids | 0.0177 |
| 215 | 14.259 | [M+H-H2O] | 161.05952 | C10H10O3 | -1.3166 | 3-Methoxycinnamic acid | Organic acids | 0.0175 |
| 216 | 7.721 | [M-H] | 861.18835 | C42H38O20 | 0.9761 | Sennoside B | Anthraquinones | 0.0175 |
| 217 | 17.608 | [M-H] | 269.04565 | C15H10O5 | 4.4767 | Genistein | Flavonoids | 0.0175 |
| 218 | 0.958 | [M+H] | 330.20609 | C20H27NO3 | -0.6784 | Hetisine | Alkaloids | 0.0170 |
| 219 | 1.128 | [M+H] | 150.05833 | C5H11NO2S | -0.1122 | L-Methionine | Others | 0.0169 |
| 220 | 14.759 | [M+H] | 425.37726 | C30H48O | -1.2562 | Lupenone | Terpenoids | 0.0166 |
| 221 | 14.583 | [M+H] | 256.26303 | C16H33NO | -1.7959 | Hexadecanamide | Amides | 0.0163 |
| 222 | 15.7 | [M-H-H2O] | 471.34808 | C30H48O4 | -2.0752 | Maslinic acid | Terpenoids | 0.0159 |
| 223 | 7.657 | [M+H] | 465.10242 | C21H20O12 | -0.7212 | Quercetin-3-O-glucoside | Flavonoids | 0.0159 |
| 224 | 15.057 | [M+H] | 391.28351 | C24H38O4 | -1.442 | Bis(2-ethylhexyl) phthalate | Organic acids | 0.0159 |
| 225 | 8.426 | [M-H] | 861.18829 | C42H38O20 | 1.1888 | Sennoside A | Anthraquinones | 0.0158 |
| 226 | 7.93 | [M+H] | 133.06465 | C9H8O | -1.052 | Cinnamaldehyde | Others | 0.0154 |
| 227 | 9.087 | [M-H] | 151.03912 | C8H8O3 | 1.0125 | 4-hydroxyphenylacetic acid | Phenolic acids | 0.0152 |
| 228 | 11.09 | [M-H] | 267.06628 | C16H12O4 | 4.1145 | Formononetin | Flavonoids | 0.0151 |
| 229 | 19.331 | [M+H] | 279.0929 | C18H15OP | -4.1074 | Triphenylphosphine oxide | Others | 0.0143 |
| 230 | 9.507 | [M-H] | 147.04417 | C9H8O2 | 0.8037 | Cinnamic acid | Phenolic acids | 0.0139 |
| 231 | 11.08 | [M-H] | 255.06625 | C15H12O4 | 4.1884 | Pinocembrin | Flavonoids | 0.0137 |
| 232 | 14.943 | [M+H] | 263.23657 | C18H32O2 | -1.4051 | octadec-9-ynoic acid | Fatty acids | 0.0137 |
| 233 | 10.581 | [M+ACN+H] | 177.12718 | C10H16O | -1.1839 | Camphor | Volatile oil | 0.0137 |
| 234 | 5.556 | [M-H] | 865.19836 | C45H38O18 | 1.0677 | Procyanidin C1 | Flavonoids | 0.0136 |
| 235 | 8.122 | [M-H] | 435.1297 | C21H24O10 | 2.5887 | Phloretin-2'-O-glucoside | Flavonoids | 0.0135 |
| 236 | 1.651 | [M-H] | 205.03481 | C7H10O7 | 2.5665 | 3-Hydroxy-3-(methoxycarbonyl)pentanedioic acid | Others | 0.0122 |
| 237 | 4.281 | [M-H] | 203.08208 | C11H12N2O2 | 2.7459 | TRYPTOPHAN | Amino acids | 0.0121 |
| 238 | 7.679 | [M+H] | 437.14386 | C21H24O10 | -1.1806 | Phlorhizin | Flavonoids | 0.0120 |
| 239 | 9.426 | [M-H] | 209.11784 | C12H18O3 | 2.9778 | Jasmonic acid | Fatty acids | 0.0120 |
| 240 | 9.935 | [M+NH4] | 151.11172 | C10H14O | -0.454 | Perillene | Terpenes | 0.0118 |
| 241 | 9.161 | [M-H] | 181.04993 | C9H10O4 | 2.1623 | 4-O-Methylphloracetophenone | Volatile oil | 0.0118 |
| 242 | 3.221 | [M-H] | 109.02836 | C6H6O2 | 0.0543 | Catechol | Phenolic acids | 0.0117 |
| 243 | 11.761 | [M-H] | 205.15933 | C14H22O | 3.1217 | 2,4-di-tert-Butylphenol | Volatile oil | 0.0116 |
| 244 | 6.976 | [M+H] | 451.12283 | C21H22O11 | -1.4511 | Eriodictyol-7-O-glucoside | Flavonoids | 0.0111 |
| 245 | 14.143 | [M+H] | 307.26251 | C20H34O2 | -2.098 | Linolenic acid ethyl ester | Fatty acids | 0.0111 |
| 246 | 8.589 | [M-H] | 191.0343 | C10H8O4 | 2.1805 | 7,8-Dihydroxy-4-methylcoumarin | Coumarins | 0.0109 |
| 247 | 10.856 | [M+H] | 233.11708 | C14H16O3 | -0.7036 | Fraxinellone | Sesquiterpenoids | 0.0107 |
| 248 | 18.495 | [M-H] | 452.2782 | C21H44NO7P | 2.7 | Glycerophospho-N-palmitoyl ethanolamine | Others | 0.0104 |
| 249 | 15.906 | [M+H] | 340.35684 | C22H45NO | -1.6146 | Docosanamide | Amides | 0.0100 |
| 250 | 14.54 | [M-H] | 227.2012 | C14H28O2 | 3.3125 | Myristic acid | Fatty acids | 0.0100 |
| 251 | 7.387 | [M-H] | 167.03416 | C8H8O4 | 1.6716 | Orsellinic acid | Fatty acids | 0.0100 |
| 252 | 4.353 | [M-H] | 163.0392 | C9H8O3 | 1.4059 | 2-hydroxycinnamic acid | Organic acids | 0.0096 |
| 253 | 5.563 | [M+H] | 325.09158 | C15H16O8 | -1.2241 | skimmin | Coumarins | 0.0094 |
| 254 | 16.792 | [M+H] | 427.39301 | C30H50O | -1.0233 | 3-Epilupeol | Terpenoids | 0.0093 |
| 255 | 7.453 | [M-H] | 465.14053 | C21H24O9 | 3.0002 | 4-Deoxyphloridzin | Flavonoids | 0.0093 |
| 256 | 10.32 | [M+H] | 387.17975 | C22H26O6 | -1.6777 | Pinoresinol dimethyl ether | Lignins | 0.0092 |
| 257 | 5.06 | [M+H] | 165.05457 | C9H8O3 | -0.3347 | 3-Hydroxycinnamic acid | Phenolic acids | 0.0092 |
| 258 | 7.695 | [M+H] | 435.1282 | C21H22O10 | -0.8479 | Naringenin-7-O-glucoside | Flavonoids | 0.0091 |
| 259 | 13.773 | [M+H] | 251.20016 | C16H26O2 | -1.5598 | Clareolide | Terpenoids | 0.0090 |
| 260 | 6.436 | [M-H] | 131.0703 | C6H12O3 | 0.6679 | 6-Hydroxycaproic acid | Fatty acids | 0.0089 |
| 261 | 12.315 | [M+H] | 455.35168 | C30H46O3 | -0.2287 | Ursonic acid | Terpenoids | 0.0089 |
| 262 | 14.03 | [M+H-H2O] | 397.30914 | C27H42O3 | -2.4262 | Diosgenin | Terpenoids | 0.0086 |
| 263 | 15.049 | [M+H] | 284.29437 | C18H37NO | -1.4731 | Stearamide | Amides | 0.0086 |
| 264 | 5.493 | [M-H] | 263.01965 | C9H12O7S | 4.7066 | 4-Hydroxy-3- methoxyphenylglycol sulfate | Others | 0.0082 |
| 265 | 14.979 | [M-H] | 305.2486 | C20H34O2 | 3.068 | 8Z,11Z,14Z-Eicosatrienoic acid | Fatty acids | 0.0071 |
| 266 | 14.228 | [M-H] | 299.20175 | C20H28O2 | 3.9943 | Tretinoin | Others | 0.0069 |
| 267 | 7.875 | [M-H] | 239.03455 | C14H8O4 | 2.764 | 2,6-dihydroxyanthraquinone | Anthraquinones | 0.0069 |
| 268 | 5.527 | [M-H] | 319.04602 | C15H12O8 | 3.6884 | Dihydromyricetin | Flavonoids | 0.0068 |
| 269 | 9.247 | [M-H+HAc] | 447.0936 | C21H20O11 | -1.2111 | Luteolin-7-O-glucoside | Flavonoids | 0.0066 |
| 270 | 5.938 | [M-H] | 449.10934 | C21H22O11 | 3.3548 | isookanin-7-O-glucoside | Flavonoids | 0.0065 |
| 271 | 9.874 | [M-H] | 182.04518 | C8H9NO4 | -0.6762 | 4-Pyridoxic acid | Organic acids | 0.0064 |
| 272 | 14.785 | [M-H] | 241.21706 | C15H30O2 | 3.5222 | Pentadecanoic acid | Fatty acids | 0.0064 |
| 273 | 7.436 | [M-H] | 144.04457 | C9H7NO | 0.9356 | 4-Hydroxyquinoline | Alkaloids | 0.0063 |
| 274 | 15.662 | [M-H] | 335.29572 | C22H40O2 | -3.9256 | 13Z,16Z-Docosadienoic Acid | Fatty acids | 0.0063 |
| 275 | 7.881 | [M-H] | 449.10922 | C21H22O11 | 3.083 | Okanin-4'-O-glucoside | Flavonoids | 0.0062 |
| 276 | 8.893 | [M+H-H2O] | 147.08044 | C10H12O2 | -0.9604 | EUGENOL | Phenylpropanoids | 0.0060 |
| 277 | 3.169 | [M-H] | 174.05533 | C10H9NO2 | 2.1449 | Indole-3-acetic acid | Alkaloids | 0.0058 |
| 278 | 7.047 | [M-H] | 245.09314 | C13H14N2O3 | 4.4386 | N-acetyltryptophan | Alkaloids | 0.0054 |
| 279 | 7.217 | [M-H] | 955.10406 | C41H32O27 | -0.6877 | Chebulinic acid | Phenolic acids | 0.0050 |
| 280 | 4.724 | [M-H] | 187.03937 | C11H8O3 | 2.6941 | Plumbagin | Anthraquinones | 0.0050 |
| 281 | 12.004 | [M-H] | 821.39648 | C42H62O16 | 3.2375 | Diammonium glycyrrhizinate | Alkaloids | 0.0049 |
| 282 | 15.334 | [M+H] | 397.34622 | C28H44O | -2.0635 | Vitamin D2 | Others | 0.0047 |
| 283 | 9.996 | [M+H] | 369.1178 | C17H20O9 | -0.3986 | methyl chlorogenate | Others | 0.0043 |
| 284 | 13.045 | [M-H] | 469.33249 | C30H46O4 | 2.669 | 18-β-Glycyrrhetinic acid | Terpenoids | 0.0041 |
| 285 | 11.495 | [M-H] | 241.14429 | C13H22O4 | 3.0248 | (Z)-2-octylpent-2-enedioic acid | Alkaloids | 0.0040 |
| 286 | 5.944 | [M-H] | 159.06532 | C7H12O4 | 0.8647 | Pimelic acid | Fatty acids | 0.0039 |
| 287 | 3.285 | [M-H] | 153.05484 | C8H10O3 | 1.5896 | 3,4-Dihydroxyphenyle+G288thanol | Phenolic acids | 0.0038 |
| 288 | 9.99 | [M-H] | 577.1564 | C27H30O14 | 1.3219 | Apigenin-7-O-neohesperidoside | Flavonoids | 0.0037 |
| 289 | 7.161 | [M+FA-H] | 223.06075 | C11H12O5 | -0.9698 | Sinapic acid | Phenolic acids | 0.0036 |
| 290 | 4.37 | [M-H] | 179.03412 | C9H8O4 | 1.838 | Acetylsalicylic acid | Organic acids | 0.0032 |
| 291 | 0.802 | [M-H] | 145.06081 | C5H10N2O3 | 0.0021 | Glutamine (D) | Others | 0.0027 |
| 292 | 0.783 | [M-H] | 154.0614 | C6H9N3O2 | 1.9364 | L-Histidine | Amino acids | 0.0019 |
